# Tocopheryl acetate 20% spray for elimination of head louse infestation: a randomised controlled trial comparing with 1% permethrin creme rinse

**DOI:** 10.1186/2050-6511-14-43

**Published:** 2013-09-03

**Authors:** Ian F Burgess, Nazma A Burgess, Elizabeth R Brunton

**Affiliations:** 1 Medical Entomology Centre, Insect Research & Development Limited, Cambridge, UK

**Keywords:** Head lice, Pediculosis, Medical device, Medicinal product, Permethrin, Tocopheryl acetate, Vitamin-E

## Abstract

**Background:**

Tocopheryl acetate is viscous oily fluid used in a range of preparations for skin and scalp care in Italy. Observational and *in vitro* data have suggested a high level of efficacy against head louse infestation. The purpose of this investigation was to confirm the activity of tocopheryl acetate in a clinical setting in comparison with a standard widely used preparation.

**Methods:**

A spray formulation containing tocopheryl acetate 20% in cyclomethicone was compared with permethrin 1% creme rinse for treatment of head louse infestation in a randomised, assessor blind, trial. Forty-five people were treated on two occasions 7 days apart. The spray was applied to dry hair for 20 minutes then washed. Participants treated with permethrin washed their hair and towel dried it before treatment for 10 minutes. Assessments were made by dry detection combing 1, 6, 9, and 14 days after first treatment.

**Results:**

The tocopheryl acetate 20% spray was significantly (p = 0.033) more effective than permethrin 1%, using intention to treat worst case analysis, in which there were 13/23 (56.5%) successful treatments for tocopheryl acetate compared with 5/22 (22.7%) for permethrin. After unprecedented issues of re-infestation within households had been taken into account the underlying cure rate was 17/23 (73.9%) for tocopheryl acetate compared with 5/22 (22.7%), Odds Ratio 9.63 (95% CI, 2.46 to 37.68) (p < 0.001).

**Conclusions:**

The tocopheryl acetate spray was significantly more effective than the permethrin product, was cosmetically acceptable, and not affected by current problems with resistance.

**Trial registration:**

Current Controlled Trials ISRCTN45553737.

## Background

Tocopheryl acetate, a vitamin-E ester, has long been used as a skin emollient and anti-oxidant in a variety of cosmetic and cosmeceutical preparations. Tocopheryl acetate has limited applications on its own because it is a high viscosity (>700 centistokes (cSt)), high surface tension (0.0337 newtons/metre (N/m)) oil, and is therefore normally formulated in more fluid materials such as decamethylcyclopentasiloxane, a low surface tension (0.018 N/m), low viscosity (2.4 cSt) silicone solvent. This type of mixture is capable of delivering small quantities of the active substance to target sites that would otherwise be inaccessible to the viscous fluid.

Fluid medical devices based on synthetic oils are widely used in Europe, Australia, and South America to control head lice and are generally claimed to block the respiratory structures of the insects [1-4]. However, increasing numbers of consumers express an interest to use naturally derived materials rather than mineral oils. Relatively few fixed plant oils have been shown to have activity and some are relatively unstable or have only been tested in developing countries [5,6], which may explain why, in several European countries, the use of neurotoxic insecticides against head lice persists despite evidence that many of the products are no longer effective.

Tocopheryl acetate in various formulations is used for a variety of protective or preventive barrier applications on inflamed or dry skin and in the spray dosage form was also designed to be suitable for application to the hair and scalp as a general conditioning agent. This use was incidentally seen to have novel potential as an anti-head louse agent. As a preliminary to investigating clinical control of head lice using the 20% tocopheryl acetate spray, we ran a series of *ex vivo* tests against head lice, which confirmed that 20% tocopheryl acetate completely immobilises lice and inhibits the hatching of louse eggs in a similar way to dimeticone based products [7]. As a result a class I medical device based on this activity was registered in Italy in 2010.

Here we report a clinical investigation conducted using the methodologies underpinning the recently published guidelines for trials of pediculicides [8]. The study compared a patented tocopheryl acetate 20% spray with an insecticide product based on 1% permethrin, the most potent of the pyrethroid insecticides used against lice.

## Methods

### Objective and participants

Participants were recruited to the study in essentially the same way as used for previous trials by advertising on local radio and by direct contact with previous study participants who had expressed an interest in taking part in further research. Potential participants were supplied with an information booklet prior to recruitment, and an enrolment visit arranged a minimum of 24 hours later.

We screened all assenting members of each household for presence of live lice by using a plastic detection comb (“PDC” comb, KSL Consulting, Helsinge, Denmark) on dry hair. Each infested member could be enrolled if: older than 6 months; had not been treated with head louse products during the previous 2 weeks; or treated with trimethoprim containing products during the previous 4 weeks. We also excluded those who had used permanent waves or hair colours during the previous 4 weeks because some products in these categories still use thiol-based ingredients that may exert an insecticidal effect, particularly against louse eggs, and some prospective participants had used them for this purpose in the past. We asked all prospective participants to confirm that they were unaware of allergy or sensitivity to pyrethroid insecticides or any other ingredient of the test products. Adult females also confirmed they were not breast feeding or pregnant, and using adequate contraception. We excluded people with long-term scalp conditions other than head louse infestation, earlier participants in this trial, and anyone who had participated in another clinical study within 1 month before screening.

At the first visit we also collected baseline demographic data on gender, age, hair characteristics, and previous pediculicide use. All treatments and assessment visits were domiciliary and no payment was offered for participation. Any ineligible people infested with lice were offered a standard of care treatment (4% dimeticone liquid gel) to minimise the risk of reinfestation of study participants.

### Ethics

Ethical approval for the study was granted by NRES Committee South Central - Berkshire B (EudraCT 2011-001892-38).

The study was conducted in conformity with the principles of the Declaration of Helsinki, of European Union Directive 2001/20/EC, and of the ICH Topic E11 guideline. All participants stated before giving consent that they had read the participation information booklet (PIB) and understood the purpose and requirements of the study. Parents or guardians gave written consent for children younger than 16 years. Children also provided written or verbal assent, according to age, witnessed by the parent or guardian.

### Treatment

The products were different both physically and in the method of application, so this study was single blinded with post-treatment assessments performed by investigators unaware of which treatment products had been used (assessor blinded). The products were applied following the instructions for use given by the manufacturers.

Tocopheryl acetate 20% spray (LiceKO^®^, Panin S.r.l, Rovigo, Italy) is made from allergen free, nature identical, tocopheryl acetate dissolved in decamethylcyclopentasiloxane (cyclomethicone) in a 20:80 ratio [7]. It was supplied in 100 mL bag-in-can sprays, i.e. the pressurised air propellant gas is not mixed with the product so pH stabilisation is not an issue. We applied the spray directly to dry hair, massaging it in with fingers, working systematically around the scalp to ensure even coverage. The treatment time was 20 minutes followed by washing with shampoo and rinsing out.

Permethrin 1% creme rinse (Lyclear**^®^** creme rinse, Omega Pharma UK, London, UK) is made from 1% 25:75 cis:trans permethrin in a conditioning base containing 20% propan-2-ol as a co-solvent. It was supplied in 59 mL plastic bottles with a flip cap dispenser. It was applied after washing the hair with a non-medicated shampoo followed by towel drying. We applied the rinse a few millilitres at a time and massaged in to thoroughly coat the hair and scalp. It was left in place for 10 minutes followed by rinsing out with water. Parents/care givers performed the hair washing and rinsing.

The first treatment day was designated Day 0. We repeated the treatments 7 days later. We asked participants not to use nit combs or other louse treatments during the course of the study.

### Outcome measures

The primary outcome measure for the study was elimination of lice after two applications of product. We assessed efficacy by dry detection combing on Days 1 and 6, before the second treatment, then on Days 9 and 14. Any lice found were collected, fixed into the case record, and examined by microscope to determine the development stage. A successful treatment was defined as no lice found on Days 9 and 14.

Outcomes of treatment were classified as cure, reinfestation following cure, or treatment failure.

### Sample size

We designed the study so we could detect superior activity of either product. We assumed that all participants were independent. Although there is ample evidence of widespread resistance to permethrin it is still used as a benchmark for comparison with novel treatments. Consequently, we expected a considerable difference in outcome between the two treatments and, using previously reported outcomes for permethrin [9-11], we estimated a success rate of about 30%. *Ex vivo* data for tocopheryl acetate indicated a potential for complete success [7], but such outcomes are rarely encountered in practice so our estimate for clinical success was likely to be of the order of 90%.

We estimated a sample size of 44 participants (22 per group) had more than 95% confidence and 90% power to detect a difference in the success rates of 60% between the two products, following the second treatment. The sample size made allowance for dropout. This sample size was also able to detect a difference of 42% with 90% confidence and 80% power.

### Randomisation and blinding

The randomised treatment allocation sequence was derived using an online computer generated list from http://www.randomization.com (seed 10028, 23^rd^ September 2011). We used instruction sheets enclosed in opaque, sealed, sequentially numbered envelopes, distributed to investigators in balanced blocks of 8, to hide the allocation until the point of delivery. A copy of the listing was prepared in case an emergency code break was required. Investigators allocated the numbered envelopes in sequence. Randomisation was by individual so different family members could receive different treatments.

### Statistics and outcome measures

Statistical analyses were performed blind of treatment allocation. Fisher exact tests were used for presence/absence variables. Differences in success rates were measured by the 95% confidence interval calculated using a normal approximation to the binomial distribution. Quantitative variables were compared by using the Mann–Whitney U test. We conducted analyses using Oxstat II, version 1.1, EpiInfo, version 6, and purpose built calculators for normal approximation and Kruskal-Wallis/Mann–Whitney tests.

## Results

### Participants

We performed this study in Cambridgeshire, UK. Between 10^th^ October 2011 and 2^nd^ February 2012, we screened 75 people for head lice and enrolled 45 participants in the study from 28 households. A further 60 household members either declined screening, were ineligible, or were unavailable. Enrolled people comprised 39 children and 6 adults aged between 2 and 45 years, median 9 years. Demographic characters of this study population showed no significant differences from populations enrolled in recent previous studies [9-12]. The proportion of participants with heavier infestations at enrolment was also similar to previous studies.

**Figure 1:**
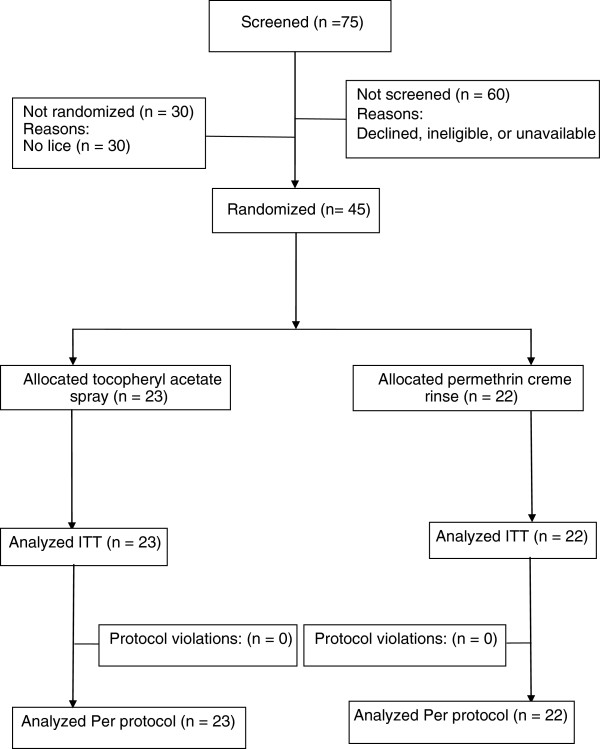
Flowchart of participants enrolled in the study.

We enrolled 10 households with more than one participating family member: 7 families having two, and one family each for three, four, and six participants. The most common household sizes were five (8 houses), four (7 houses), three and six (6 houses) each. We planned for equal numbers of people receiving each treatment (22 per group) with a final enrolment of 23 receiving tocopheryl acetate 20% spray and 22 receiving permethrin 1% creme rinse. All 45 participants completed all study assessments and there were no instances of non-compliance. Consequently, all participants were included in both the intention to treat (ITT) and per-protocol (PP) analyses (Figure 1).

The main endpoint analysis was the comparison of rate of cure in the 45 participants in the ITT population. According to this criterion, using worst case analysis, tocopheryl acetate 20% spray performed significantly better (p = 0.033, 95% confidence interval (CI), 0.05 to 0.62) with success achieved for 13/23 (56.5%) of the participants compared with 5/22 (22.7%) of those treated with permethrin 1% creme rinse, a difference of 33.8% (Odds Ratio (OR) 4.42, 95% CI, 1.21 to 16.12).

The reason we performed a worst case analysis is because randomisation in this study produced an unusually high proportion of households in which at least two members received different treatments. The result was that tocopheryl acetate eliminated lice from several people who were then reinfested from family members receiving the much less effective permethrin. When we analysed the lice collected during post-treatment assessments it indicated a higher actual treatment success rate for tocopheryl acetate of 17/23 (73.9%), without a similar change in the outcome for permethrin, a difference of 51.2% (OR 9.63, 95% CI, 2.46 to 37.68). This analysis also revealed that the 20% tocopheryl acetate spray was not able to prevent all louse eggs from hatching because small numbers of newly emerged nymphs were found on 14 participants between the first and second treatments. Six participants (26.1%) also had newly emerged nymphs following the second application of spray, showing that some eggs may take longer than 7 days to hatch.

Although we found lice in both groups before the second treatment on Day 7, applying the spray a second time eliminated the majority of infestation. After the second treatment, people in the tocopheryl acetate 20% group had significantly (p < 0.05) fewer lice of any development stage except adult females at Day 9, and adults and third stage nymphs at Day 14. These adult lice and older nymphs, which had migrated from infested family members treated with permethrin, reduced the overall efficacy outcome for tocopheryl acetate from 73.9% to 56.5%. We also analysed the distribution of outcome and success rates for subsets of the population by various factors: sex, age, level of initial infestation, hair length, hair thickness, hair curl, and hair type. Additional tests were made for the significance of variations in the rates of cure or re-infestation by levels of these factors and the results are summarised in Table 1.

**Table 1:** Rates of cure overall and by data subgroup - ITT population

		**Success rate**				**p value**
		**Tocopheryl acetate 20%**		**Permethrin 1%**		
		**n/N**	**%**	**n/N**	**%**	
Whole population						
Elimination of initial infestation		17/23	73.9	5/22	22.7	<0.001
Worst case analysis		13/23	56.5	5/22	22.7	0.033
Data subgroup						
Sex	- males	1/2	50.0	1/3	33.3	NS
	- females	12/21	57.1	4/19	21.1	0.027
Age	- 2 to 9	10/13	76.9	4/11	36.4	NS
	- 10 to 15	1/7	14.3	0/8	0.0	NS
	- 16+	2/4	50.0	1/3	33.3	NS
Infestation	- light	9/12	75.0	3/11	27.3	0.039
	- moderate	2/4	50.0	2/5	40.0	NS
	- heavy	2/7	28.6	0/5	0.0	NS
Hair length	- above ears	1/2	50.0	1/3	33.3	NS
	- ears to shoulders	5/9	55.6	1/7	14.3	NS
	- below shoulders	7/12	58.3	3/12	25.0	NS
Hair thickness	- fine	3/4	75.0	2/4	50.0	NS
	- medium	4/6	66.7	1/6	16.7	NS
	- thick	6/13	46.2	2/12	16.7	NS
Hair curl	- straight	4/11	36.4	4/14	28.6	NS
	- wavy	6/8	75.0	0/6	0.0	<0.01
	- curly	3/4	75.0	1/2	50.0	NS
Hair type	- dry	1/2	50.0	1/2	50.0	NS
	- normal	11/19	57.9	5/18	27.8	NS
	- greasy	1/2	50.0	0/2	0.0	NS

Given the small sample size, it was not surprising that few differences or trends were significant at p < 0.05. However, we did find significant trend differences (p < 0.05) between the treatment groups for females, and those with a “light” infestation at enrolment, and in the populations overall. The most highly significant difference of outcome was in those with “wavy” hair (p < 0.01), possibly due to the quantity of preparation that could be held by the hair, which would have influenced the level of contact between the product and the lice and louse eggs. Nearly all (20/23) people treated with tocopheryl acetate and their carers expressed a preference for using the spray compared with the permethrin creme rinse with respect to comfort and convenience, as well as success of outcome.

We weighed the containers of product before and after use to measure product usage. Slightly more treatment was applied on the first occasion than the second, with a mean application of 39.5 g of spray. In contrast the mean quantity of creme rinse used was nearly double for each treatment at 76.3 g, from which the cost of a single treatment application proved to be similar for each product at approximately €6.50 to €6.85.

### Adverse events

There were 13 adverse events in 13 participants, of which 11 were simple accidents or childhood infections not related to treatment. One adverse event in each group was an application site event of stinging or itching considered to have some relationship to treatment or the washing process involved in removing the treatment. In the case of permethrin creme rinse, stinging occurred during the treatment application process, indicating irritation of existing excoriations by the treatment. In the case of tocopheryl acetate spray, itching occurred during the washing off process that was possibly associated with the shampoo used.

## Discussion

We have found that tocopheryl acetate, applied as a 20% spray, was significantly more effective to eliminate head lice than the insecticide permethrin. We have also found evidence that resistance to pyrethroid insecticides like permethrin has not diminished in the study area despite reduced use of the insecticide during the past few years. However, the presentation of resistance in the UK is largely different from that in some other countries where susceptibility to pyrethroids may be reduced, but not eliminated, and resistance to alternative insecticides like malathion is either not found or is of limited impact [13,14]. Although knockdown resistance (*kdr*) is present in the country it appears to play only a minor role in treatment failure compared with a double resistance to permethrin and malathion mediated by elevated levels of non-specific esterases [Burgess IF, unpublished] [15]. As a result, the impact of resistance has continued to reduce efficacy of insecticide based products to the point that they are now effectively useless for many consumers [9-12,16].

The mode of action of tocopheryl acetate to kill head lice is currently unconfirmed but, as it is hydrophobic and has a high viscosity (>700 cSt), we believe it has an occlusive effect on the louse surface and blocks the spiracular structures in a similar manner to the high molecular weight dimeticone used in some of the most widely used products in Europe [3].

Since 2005, physically acting medical device products for control of head louse infestation have increased in number, availability, and variety of dosage form and have largely replaced insecticides in several countries in Europe, and have a large market share in Australia and New Zealand. However, clinical data are only available in the public domain for a limited number of these products. This treatment concept is generally popular with consumers because the products do not contain insecticides and most do not contain irritating solvents or preservatives. But comments from consumers indicate that some are not effective and others are difficult to use.

In recent times an additional problem for all families attempting to eliminate head louse infestation has been reinfestation. Irrespective of the effectiveness of the product they are using there is no protection after washing the treatment off so the children could potentially be reinfested from the next contact with their friends. Fortunately in most households everyone is normally treated using the same product so that everyone is usually either cured or not at the same time. In clinical studies randomised by individual it is often the case that some households have different members receiving different treatments. For most medications this does not constitute an issue but for head louse studies, if there is a disparity of efficacy, those receiving the more effective product are at continual risk of reinfestation from those on the less effective one. This is an issue we have addressed in the past by development of an algorithm to identify where the few cases of reinfestation occur [9-12,16]. However, in this study a high proportion of households had people in receipt of different treatments, and most people receiving permethrin still had lice after treatment. As a result it was inevitable that any siblings who had been treated with tocopheryl acetate would be at risk of reinfestation, which was identified by being found to be free from lice after treatment but infested again by adult lice and third stage nymphs by the end of the study. Of course, it is impossible to confirm absolutely that the reinfestation arose from such contacts, which is why we conducted a worst case analysis also. Perhaps for this reason it would be wiser to consider randomisation by household in future studies but this has drawbacks because it requires larger numbers of participants, which could make the cost of investigations prohibitive for small companies, and has hitherto not been favoured by some regulatory agencies who have preferred randomisation by individual because each person is seen as being independent [12].

Fixed vegetable oils, such as olive and coconut oils, have been used as hair grooming agents for centuries in several cultures while others such as neem have been used to treat infections and infestations. Whether they have any real activity against head lice is unknown but modern folklore from the USA, and other western cultures where these oils are not routinely used, suggests they occlude the louse respiratory tract, similar to the activity of synthetic oils. However, with a low viscosity of the order of 80–90 cSt combined with a low surface tension, these oils would form a thin film on lice and hairs that could only act slowly and may not deliver sufficient material to the respiratory structures of the lice. Certainly, in geographic areas where these vegetable oils are routinely used on hair, lice are common and appear to survive unhindered by the oils. Neem seed oil, which is marketed as a shampoo formulated material, is reported to demonstrate a rapid activity, killing lice within a few minutes [5,6]. Such speed of effect is unlikely to occur by occlusion alone because neem oil is chemically similar to extra virgin olive oil but with the addition of small quantities of a number of complex terpenoids. The reported rapid activity of neem shampoo either suggests a pharmacological activity, which would place the preparations into the sphere of medicinal products rather than devices, or else the activity must be due to formulation components other than neem such as powerful surfactants stripping the lipid from the louse surface. Similarly, eucalyptus and tea tree oils, as well as other monoterpene based products, are reported to show good efficacy [4,9,17-19] but these oils have a recognised activity to inhibit acetylcholine esterase and other enzymes [20], which would make them pharmacologically active also.

Tocopheryl acetate is different from other products based on natural materials because it has a high viscosity causing it to adhere to lice or hair without absorption across the insect cuticle. As a 20% spray in a low viscosity carrier, tocopheryl acetate becomes an effective physical occlusive agent that is not easily removed from the hydrophobic louse cuticle by shampoo. Consequently, even a short application time of 20 minutes proved effective because the immobilised lice remain coated with the active material. As a preparation we have found it to be of similar efficacy to other widely accepted physically acting preparations but with the added appeal for consumers that it is “naturally” based. Like the original cosmetic tocopheryl acetate spray upon which this product was based (Vea^®^ Spray, Hulka S.r.l., Rovigo, Italy), it has the added effect to reduce inflammation of the scalp resulting from previous louse bites, thus reducing irritation and dryness. Future investigations will look at ways to limit flow of the sprayed fluid along the hair shafts, which should reduce the quantity of product required for an adequate treatment and improve the performance of the product overall, particularly against louse eggs.

## Conclusion

In this randomized, controlled, assessor-blinded, clinical investigation we have shown that a 20% tocopheryl acetate based spray (LiceKO^®^ spray) was significantly more effective than a 1% permethrin creme rinse. The product was both better tolerated and more cosmetically acceptable.

## Competing interests

IFB is a consultant to several major and minor manufacturers of pediculicides, medical devices, and combs for use against head lice and their eggs. The other authors declare they have no competing interests in this work.

## Author contributions

All three authors contributed towards the design of the study. Participant enrolment was performed by IFB and ERB and the majority of outcome data were acquired by NAB. ERB managed the study documentation and IFB conducted the analyses and drafted the manuscript. All three authors agreed the manuscript.

## Pre-publication history

The pre-publication history for this paper can be accessed here:

http://www.biomedcentral.com/2050-6511/14/43/prepub
